# Efficacy and Safety of Acupuncture Therapy for Patients with Acute Ankle Sprain: A Systematic Review and Meta-Analysis of Randomized Controlled Trials

**DOI:** 10.1155/2020/9109531

**Published:** 2020-10-16

**Authors:** Ai-Feng Liu, Shu-Wei Gong, Ji-Xin Chen, Jing-Bo Zhai

**Affiliations:** ^1^Department of Orthopaedic Surgery, First Teaching Hospital of Tianjin University of Traditional Chinese Medicine, Tianjin, China; ^2^Graduate College, Tianjin University of Traditional Chinese Medicine, Tianjin, China; ^3^Institute of Traditional Chinese Medicine, Tianjin University of Traditional Chinese Medicine, Tianjin, China

## Abstract

**Background:**

The efficacy of acupuncture for acute ankle sprain (AAS) is controversial. This study aimed to critically assess the efficacy and safety of acupuncture for AAS.

**Methods:**

Parallel-group randomized controlled trials (RCTs) were included regardless of language or publication date. Participants with AAS were included regardless of age, sex, race, nationality, or diagnostic criteria for AAS. Experimental interventions included acupuncture alone or in combination with traditional therapies. Control interventions included no treatment, placebo, or traditional therapies. The primary outcome was the Kofoed ankle score. The secondary outcomes included visual analogue scale, duration of pain, use of painkiller, ankle circumference, effective rate, cure rate, and adverse events. PubMed, Embase, Cochrane Library, Web of Science, China National Knowledge Infrastructure, Wanfang Digital Periodicals, and Chinese Science and Technology Periodicals database were searched to identify potentially eligible studies from inception to September 10, 2020. World Health Organization International Clinical Trials Registry Platform (WHO ICTRP), ClinicalTrials.gov, Chinese Clinical Trial Registry (ChiCTR), and the reference list of eligible RCTs were checked to identify ongoing or unpublished studies. Risk of bias was assessed by the Cochrane Collaboration's tool. Statistical analyses were performed by RevMan 5.3 software. *P* < 0.05 indicated statistical significance.

**Results:**

Seventeen eligible studies were included for the statistical analysis. There was no statistically significant difference of Kofoed ankle score between acupuncture and Rest, Ice, Compression, and Elevation (RICE) group (*P*=0.75). However, acupuncture could significantly relieve pain (*P*=0.02) and increase cure rate (*P*=0.004) compared with RICE. Moreover, acupuncture plus RICE could also significantly relieve pain (*P* < 0.00001) and increase cure rate (*P*=0.01) compared with RICE alone. Acupuncture combined with massage could significantly relieve pain (*P*=0.04) compared with massage alone. Acupuncture plus Chinese medicine might be more effective for relieving pain (*P* < 0.00001), reducing the duration of pain (*P* < 0.00001), and increasing cure rate (*P*=0.0002) compared with Chinese medicine alone. Two studies reported no adverse reactions. One study reported that a participant suffered from mild drug-related allergic reaction and was healed without treatment.

**Conclusions:**

The findings of the present study suggest that acupuncture may be beneficial for AAS. However, more large-scale and well-designed RCTs are warranted.

## 1. Introduction

Acute ankle sprain (AAS) is defined as an acute injury of the ankle ligament [1]. Ankle sprain is one of the major injuries among the general population and athletes [2–4]. AAS may result in acute pain, swelling, high cost, chronic ankle instability, etc. [5, 6]. There are a variety of therapeutic interventions for AAS, involving pharmacological therapies (e.g., nonsteroidal anti-inflammatory drugs) and nonpharmacological therapies (e.g., functional support, exercise, and manual mobilization) [3, 4]. However, no optimal therapies were recommended for treating AAS according to a latest evidence-based clinical guideline [4].

Acupuncture belongs to complementary and alternative medicine and is commonly used for relieving acute and chronic pain [7, 8]. Two previous systematic reviews assessed the efficacy of acupuncture for ankle sprain [1, 9]. However, the evidence on acupuncture for ankle sprain still remains inconclusive because of large heterogeneity [4]. There are some methodological flaws in the two previous studies [1, 9]. For example, high clinical heterogeneity might be caused by combining results from studies involving patients with acute and chronic ankle sprain. Different types of acupuncture resulted in the heterogeneity of interventions. Moreover, some new trials of acupuncture for AAS have been published in recent years [10–17]. However, the evidence has not been critically assessed. Therefore, we conducted an updated systematic review to assess the efficacy and safety of acupuncture for AAS.

## 2. Methods

This systematic review was registered on PROSPERO (no. CRD42020156280). It was conducted in compliance with the Preferred Reporting Items for Systematic Reviews and Meta-Analyses (PRISMA) statement [18].

### 2.1. Inclusion and Exclusion Criteria

#### 2.1.1. Types of Studies

Parallel-group randomized controlled trials (RCTs) were included regardless of language or publication date.

#### 2.1.2. Types of Participants

Participants with AAS were included regardless of age, sex, race, nationality, or diagnostic criteria for AAS.

#### 2.1.3. Types of Interventions

The experimental interventions included acupuncture alone or in combination with traditional therapies. The control interventions included no treatment, placebo, or traditional therapies. Traditional therapies for acute ankle sprain involve nonsteroidal anti-inflammatory drugs, Rest, Ice, Compression, and Elevation (RICE), functional support, exercise, manual mobilization, etc. There were no restrictions on frequency or duration of acupuncture. The following comparisons were considered if possible: (1) acupuncture alone versus no treatment/placebo/traditional therapies; (2) acupuncture plus traditional therapies versus traditional therapies alone; and (3) acupuncture plus traditional therapies versus traditional therapies plus placebo.

#### 2.1.4. Types of Outcomes

The primary outcome was the Kofoed ankle score. The secondary outcomes included visual analogue scale (VAS), duration of pain, use of painkiller, ankle circumference, effective rate, cure rate, and adverse events. Kofoed ankle score is comprised of pain, function, and mobility domain and ranges from 0 to 100 with higher score indicating less pain [19]. VAS ranges from 0 to 10 or 100 with higher score indicating more severe pain.

### 2.2. Search Strategy

Two authors (SWG and AFL) independently searched PubMed, Embase, Cochrane Library, Web of Science, China National Knowledge Infrastructure, Wanfang Digital Periodicals, and Chinese Science and Technology Periodicals database from inception to September 10, 2020, to identify potentially eligible studies. World Health Organization International Clinical Trials Registry Platform (WHO ICTRP), ClinicalTrials.gov, Chinese Clinical Trial Registry (ChiCTR), and the reference list of eligible RCTs were checked to identify ongoing or unpublished studies [20]. The detailed search strategy is available in Supplementary Material.

### 2.3. Selection of Studies and Data Extraction

All studies identified from the electronic search were imported into EndNote software. Two reviewers (SWG and AFL) independently checked the title and abstract to remove duplicates and irrelevant studies. Full texts of the remaining studies were read to identify potentially eligible studies. The selection process was summarized using a PRISMA flow diagram.

The following information was extracted independently by two reviewers (JXC and SWG). Disagreements were resolved by consensus or consultation with a third review author (JBZ).

1. Study details: title, first author, country, year of publication, design, methods of randomization, allocation, and blinding
2. Patients: age, sample size
3. Interventions: type, frequency, and duration
4. Outcome measures: Kofoed ankle score, VAS, duration of pain, use of painkiller, ankle circumference, effective rate, cure rate, and adverse events

### 2.4. Assessment of Risk of Bias

Two reviewers (JXC and SWG) independently assessed the risk of bias in eligible studies using the Cochrane Collaboration's tool [21, 22]. It includes seven important items: random sequence generation, allocation concealment, blinding of participants and personnel, blinding of outcome assessment, incomplete outcome data, selective outcome reporting, and other potential sources of bias. The risk of bias for each item was classified as low, high, or unclear. The results were presented with risk of bias graph and summary figure.

### 2.5. Statistical Analysis

Mean difference (MD) with 95% confidence intervals (CIs) was calculated for continuous variables if the same tool was used to measure a certain outcome across different studies. Otherwise, standardized mean difference (SMD) was calculated. Risk ratio (RR) with 95% CIs was used for dichotomous variables. If clinical heterogeneity was low, meta-analysis was used to estimate the overall effect. Statistical heterogeneity was evaluated by chi-square test or *I*^2^ statistics. If the *P* value of chi-square test was greater than 0.10 or *I*^2^ was less than 50%, the fixed-effect model was used to estimate the effect size. Otherwise, the random-effect model was used. The funnel plot was used to assess publication bias when at least 10 studies were included in a meta-analysis. Subgroup analyses were performed based on intervention and comparison if possible. RevMan 5.3 software was used for the statistical analysis. *P* < 0.05 indicated statistical significance. If performing meta-analysis was infeasible, a narrative description was provided.

## 3. Results

### 3.1. Literature Search

**Figure 1:**
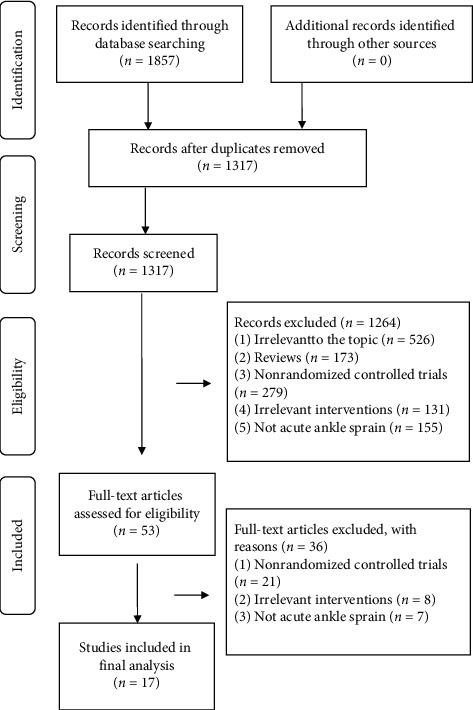
Flow diagram for study retrieval and selection.

The initial search yielded 1857 potentially eligible studies. We deleted 540 duplicates and 1264 irrelevant studies by checking the title and abstract. After reading full texts of the remaining records, 36 studies were excluded. Finally, 17 studies [10–17, 23–31] were included for the statistical analysis (Figure 1).

### 3.2. Characteristics of Included Studies

The characteristics of the included studies are summarized in Table 1. Seventeen studies involving 1528 patients were published between 1999 and 2018 in China. Sample size ranged from 20 to 90 in the experimental group and 20 to 70 in the control group. Experimental interventions included acupuncture alone or in combination with RICE, dimethyl sulfoxide, Chinese medicine, or massage. Control interventions included RICE, dimethyl sulfoxide, Chinese medicine, massage, ice and hot pack, no treatment, or infrared radiation.

### 3.3. Assessment of Risk of Bias

**Figure 2:**
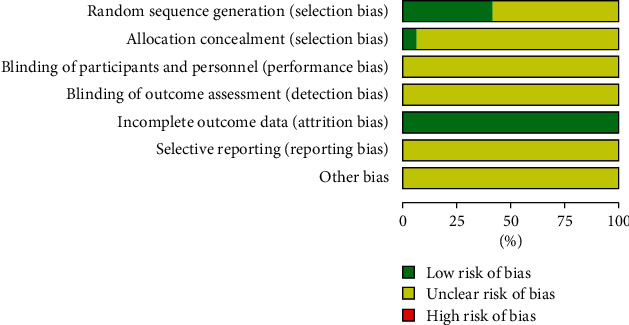
Risk of bias graph.

**Figure 3:**
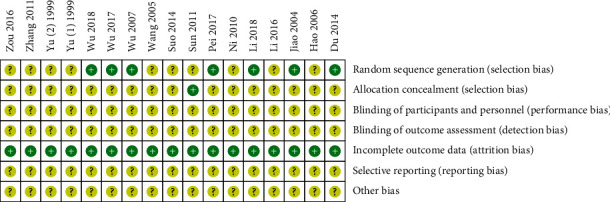
Risk of bias summary.

Risk of bias graph and summary are presented in Figures 2 and 3. Five trials [11, 14–17] used a random number table, one [25] used coin tossing, and one [28] used a computer random number generator to generate a random sequence. One study [30] used sealed envelopes to conceal allocation. Attrition bias was low because complete outcome data were reported in all included studies. Performance bias, detection bias, reporting, bias, and other bias were unclear because of insufficient information.

### 3.4. Kofoed Ankle Score

One study [12] reported the response rate of Kofoed ankle score. Response was defined as having a Kofoed ankle score greater than or equal to 75. It indicated that no statistically significant difference was found between acupuncture and RICE group (*n* = 60, RR = 1.04, 95% CI: 0.80 to 1.36, *P*=0.75).

### 3.5. Visual Analogue Scale

Narrative analyses were provided because of the heterogeneity of interventions. Wu [28] found that acupuncture could significantly relieve pain compared with no treatment (*n* = 61, MD = −6.92, 95% CI: −7.33 to −6.51, *P* < 0.00001). Li [12] found that acupuncture was superior to RICE for pain relief (*n* = 60, MD = −0.37, 95% CI: −0.67 to −0.07, *P*=0.02). Wu et al. [16] reported that acupuncture plus RICE achieved a greater level of pain relief than RICE (*n* = 90, MD = −1.14, 95% CI: −1.63 to −0.65, *P* < 0.00001). Wu et al. [15] showed that acupuncture plus massage could significantly decrease the VAS score compared with massage alone (*n* = 82, MD = −0.26, 95% CI: −0.51 to −0.01, *P*=0.04). Zou et al. [13] reported that acupuncture plus Chinese medicine could significantly relieve pain compared with Chinese medicine alone (*n* = 40, MD = −2.73, 95% CI: −2.89 to −2.57, *P* < 0.00001).

### 3.6. Duration of Pain

Li et al. [17] found that acupuncture plus Chinese medicine was more effective than Chinese medicine alone for reducing the duration of pain (*n* = 80, MD = −2.50, 95% CI: −2.97 to −2.03, *P* < 0.00001). Sun et al. [30] reported that acupuncture could significantly reduce the duration of pain compared with elastoplast (*n* = 82, MD = −3.40, 95% CI:−3.88 to −2.92, *P* < 0.00001).

### 3.7. Use of Painkillers

Wu et al. [15] found no significant difference of use of painkillers between acupuncture plus massage and massage alone (*n* = 82, RR = 0.48, 95% CI: 0.16 to 1.46, *P*=0.19).

### 3.8. Ankle Circumference

Wu et al. [15] reported that ankle circumference was not statistically different after acupuncture plus massage treatment compared with massage alone (*n* = 82, MD = −0.65, 95% CI: −1.64 to 0.34, *P*=0.20). Li [12] found that the difference of ankle circumference between the uninjured and injured ankle was not statistically different after acupuncture treatment compared with RICE (*n* = 60, MD = 0.24, 95% CI: −0.10 to 0.58, *P*=0.17).

### 3.9. Effective Rate

**Figure 4:**
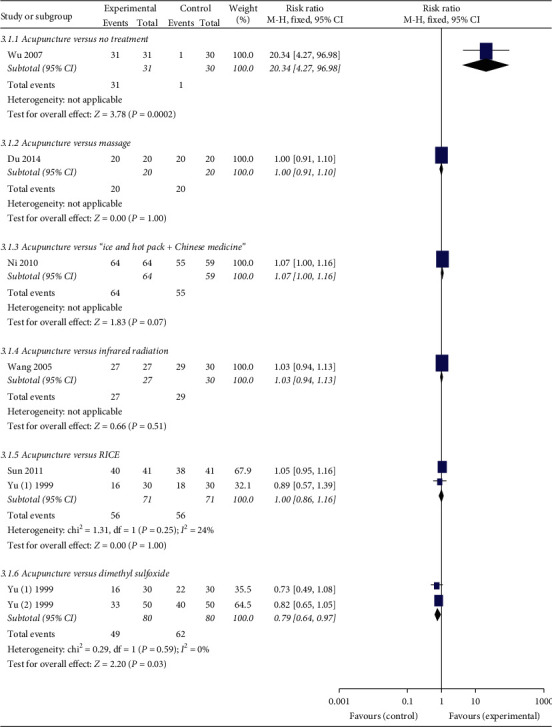
Forest plots of acupuncture versus other treatments on the effective rate.

**Figure 5:**
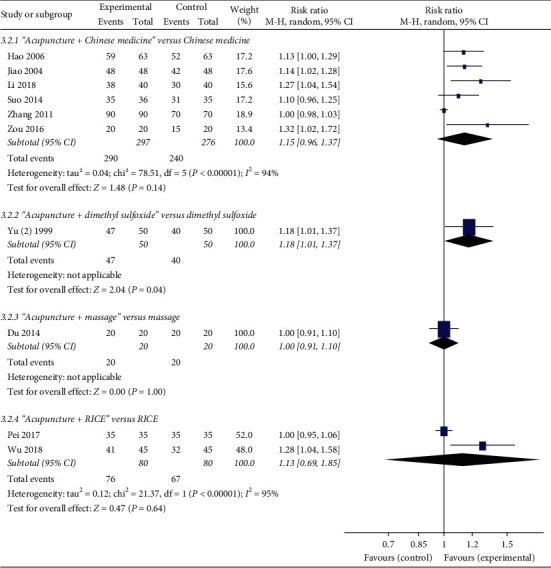
Forest plots of acupuncture plus other treatments versus other treatments on effective rate.

Fifteen studies reported the effective rate. It is defined as a ratio of the number of patients labelled as cure, excellent, or effectivity divided by the number of patients in a certain group. Wu [28] found that the effective rate in the acupuncture group was statistically higher (*P*=0.0002) than that in no treatment group (Figure 4). However, the effective rate in the acupuncture group was not different statistically from that in massage (*P*=1.00), “Ice and hot pack” plus Chinese medicine (*P*=0.07), infrared radiation (*P*=0.51), or RICE (*P*=1.00) group. A meta-analysis showed that dimethyl sulfoxide could significantly increase the effective rate compared with acupuncture (*P*=0.03). Yu [24] found that the effective rate in the acupuncture plus dimethyl sulfoxide group was statistically higher (*P*=0.04) than that in the dimethyl sulfoxide group (Figure 5). The results from meta-analyses showed that the effective rate in the acupuncture plus Chinese medicine or acupuncture plus RICE group was not different statistically from that in Chinese medicine (*P*=0.14) or RICE group (*P*=0.64). Moreover, Du et al. [11] reported that the effective rate in the acupuncture plus massage group was similar (*P*=1.00) with that in the massage group.

### 3.10. Cure Rate

**Figure 6:**
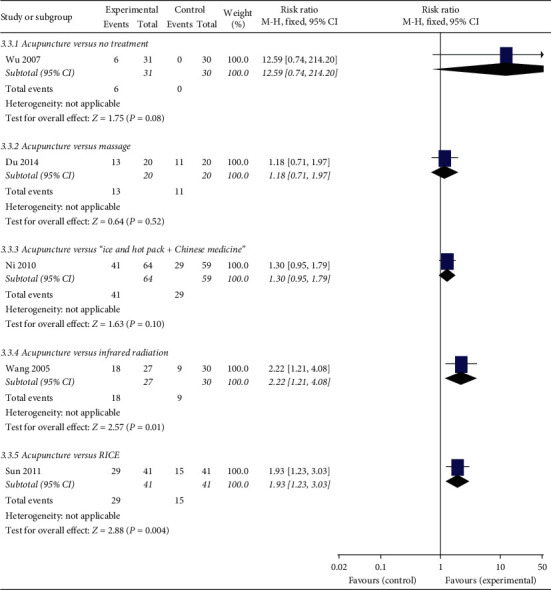
Forest plots of acupuncture versus other treatments on cure rate.

Thirteen studies reported the cure rate. It is defined as a ratio of the number of patients labelled as cure divided by the number of patients in a certain group.  Figure 6 shows that the cure rate in the acupuncture group is statistically higher than that in the no treatment (*P*=0.08), infrared radiation (*P*=0.01), or RICE (*P*=0.004) group. However, the cure rate in the acupuncture group was not statistically different from that in the massage (*P*=0.52), or “Ice and hot pack” plus Chinese medicine (*P*=0.10) group.

**Figure 7:**
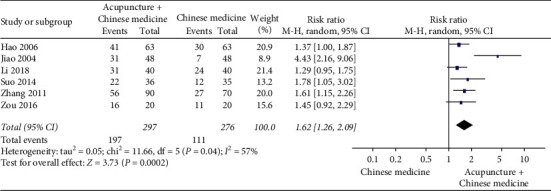
Forest plot of acupuncture plus Chinese medicine versus Chinese medicine on cure rate.

**Figure 8:**
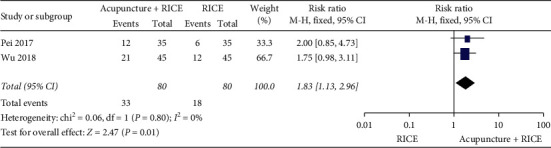
Forest plot of acupuncture plus RICE versus RICE on cure rate.

A meta-analysis showed that acupuncture plus Chinese medicine could significantly increase the cure rate (*P*=0.0002) compared with Chinese medicine alone (Figure 7). Other meta-analysis showed that acupuncture plus RICE could significantly increase the cure rate (*P*=0.01) compared with RICE alone (Figure 8). Du et al. [11] found that the cure rate in the acupuncture plus massage group was higher (*n* = 40, RR = 1.55, 95% CI: 1.00 to 2.39, *P*=0.05) than that in the massage group.

### 3.11. Adverse Events

Three included studies reported the information on adverse events. Yu [24] and Wu [28] reported no adverse reactions. Another study [23] reported that a participant suffered from mild drug-related allergic reaction and was healed without treatment.

### 3.12. Assessment of Publication Bias

No funnel plots were provided to assess publication bias because no meta-analyses involving more than ten studies were performed.

## 4. Discussion

### 4.1. Main Findings and Interpretation

The present study critically assessed the efficacy and safety of acupuncture for AAS. Overall, risk of bias assessment was limited because of incomplete reporting on risk of bias items. In view of the heterogeneity of interventions, main findings were interpreted based on comparisons between experimental and control groups.

Rest, ice, compression, and elevation (RICE) are generally used to treat acute ankle sprain in clinical practice [32]. However, a systematic review found insufficient evidence on RICE for AAS in adults [33]. RICE was also not recommended for the management of lateral ankle sprain according to a recent clinical guideline [4]. The present study found that acupuncture alone or in combination with RICE could significantly relieve pain and increase cure rate in patients with AAS compared with RICE alone. It suggests that acupuncture may be considered as a complementary and alternative therapy for treating AAS. However, more large-scale RCTs with objective outcomes are warranted to confirm these findings.

Massage belongs to nonpharmacological therapies and usually is used for the management of musculoskeletal disorders. A study found that massage might improve the flexibility and balance function of the ankle joint [34]. An evidence map showed that massage was used for treating a variety of pain conditions [35]. The present study found that acupuncture combined with massage could significantly relieve pain in patients with AAS compared with massage alone. However, the underlying mechanism of the combination for AAS is still poorly investigated.

Patients with AAS often experience acute pain and swelling associated with inflammatory reactions [36]. Previous studies showed that some Chinese medicines had anti-inflammatory properties and were used for treating musculoskeletal disorders [37–39]. The present study found that acupuncture plus Chinese medicine might be more effective for relieving pain, reducing the duration of pain, and increasing cure rate than Chinese medicine alone in patients with AAS. Nonsteroidal anti-inflammatory drugs were recommended for reducing pain and controlling swelling in patients with acute lateral ankle sprain [4]. However, the efficacy of acupuncture versus nonsteroidal anti-inflammatory drugs for AAS was not assessed because no eligible trials were identified.

The present study provided some insights into the management of AAS. Combined-modality therapy may provide additional benefits in patients with AAS. These findings may be useful for updating clinical practice guidelines. Moreover, a review reported that acupuncture could relieve pain by activating acupoints and transmitting signals to the spinal cord and brain associated with the regulation of inflammatory factors [40]. An experiment showed that acupuncture might relieve neuropathic pain in rats by inhibiting microglial activation and inflammatory responses [41]. Chen et al. [42] found that acupuncture might relieve inflammatory symptoms at the ankle joint by reducing serum TNF-*α* and anti-cyclic citrullinated peptide antibody levels. The evidence may partly explain why acupuncture may be effective for AAS.

### 4.2. Limitations

This systematic review had several limitations. Firstly, effect size might be overestimated because of small sample size. Secondly, performing the meta-analysis was limited because of the heterogeneity of interventions. Thirdly, the results should be interpreted cautiously because of methodological flaws and rarely reported objective outcomes in included studies.

## 5. Conclusions

The findings of the present study suggest that acupuncture may be beneficial for AAS. However, more large-scale and well-designed RCTs are warranted.

## Figures and Tables

**Table 1:** Characteristics of included studies.

First author	Year	Sample size (E/C)	Experimental interventions	Control interventions	Frequency of acupuncture	Duration of acupuncture	Outcomes
Yu (1) [23]	1999	30 in each group/30 in each group	Acupuncture; acupuncture + RICE(ice pack) + dimethyl sulfoxide	RICE(ice pack); dimethyl sulfoxide	Twice a day	7 days	Effective rate
Yu (2) [24]	1999	50 in each group/50	Acupuncture; acupuncture + dimethyl sulfoxide	Dimethyl sulfoxide	Not reported	7 days	Effective rate
Jiao and Wang [25]	2004	48/48	Acupuncture + Chinese medicine (shujin huoxue pill + jiejing zhitong tincture)	Chinese medicine (shujin huoxue pill + jiejing zhitong tincture)	Once a day	7 days	Effective rate, cure rate
Wang [26]	2005	27/30	Acupuncture	Infrared radiation	Once a day	5 days	Effective rate, cure rate
Hao and Wang [27]	2006	63/63	Acupuncture + Chinese medicine (herbs)	Chinese medicine (herbs)	Once two days	7 days	Effective rate, cure rate
Wu [28]	2007	31/30	Acupuncture	No treatment	Once a day	5 days	Effective rate, cure rate, VAS
Ni and Li [29]	2010	64/59	Acupuncture	Ice and hot pack + Chinese medicine	Once a day	3 days	Effective rate, cure rate
Sun and Ju [30]	2011	41/41	Acupuncture	RICE (elastoplast)	Once a day	14 days	Effective rate, cure rate, duration of pain
Zhang and Zhang [31]	2011	90/70	Acupuncture + Chinese medicine (qili powder)	Chinese medicine (qili powder)	Once a day	10 days	Effective rate, cure rate
Suo [10]	2014	36/35	Acupuncture + Chinese medicine (yunnan baiyao tincture)	Chinese medicine (yunnan baiyao tincture)	Not reported	Not reported	Effective rate, cure rate
Du [11]	2014	20 in each group/20	Acupuncture; acupuncture + massage	Massage	Once a day	3 days	Effective rate, cure rate
Li [12]	2016	30/30	Acupuncture	RICE	Once a day	7 days	VAS, Kofoed ankle score, ankle circumference
Zou [13]	2016	20/20	Acupuncture + Chinese medicine (sunshang emplastrum)	Chinese medicine (sunshang emplastrum)	Once two days	7 days	VAS, effective rate, cure rate
Pei and Wei [14]	2017	35/35	Acupuncture + RICE	RICE	Not reported	14 days	Effective rate, cure rate
Wu and Chen [15]	2017	42/40	Acupuncture + massage	Massage	Once two days	14 days	VAS, ankle circumference, use of painkiller
Wu [16]	2018	45/45	Acupuncture + RICE (plaster immobilization)	RICE (plaster immobilization)	Once two days	21 days	Effective rate, cure rate, VAS
Li [17]	2018	40/40	Acupuncture + Chinese medicine (shexiang zhuanggu emplastrum)	Chinese medicine (shexiang zhuanggu emplastrum)	Once a day	10 days	Effective rate, cure rate, duration of pain

E, experimental group; C, control group; RICE, Rest, Ice, Compression, and Elevation; VAS, visual analogue scale.

## Data Availability

All datasets presented in this study are included in the article or supplementary material.

## Supplementary Materials

Supplementary Materials Detailed search strategy is shown in the Supplementary Material.

## References

[1] Park J., Hahn S., Park J. Y., Park H. J., Lee H. (2013). Acupuncture for ankle sprain: systematic review and meta-analysis. *BMC Complement Alternative Medicine*.

[2] Fong D. T.-P., Hong Y., Chan L.-K., Yung P. S.-H., Chan K.-M. (2007). A systematic review on ankle injury and ankle sprain in sports. *Sports Medicine*.

[3] Doherty C., Delahunt E., Caulfield B. (2014). The incidence and prevalence of ankle sprain injury: a systematic review and meta-analysis of prospective epidemiological studies. *Sports Medicine*.

[4] Vuurberg G., Hoorntje A., Wink L. M. (2018). Diagnosis, treatment and prevention of ankle sprains: update of an evidence-based clinical guideline. *British Journal of Sports Medicine*.

[5] Doherty C., Bleakley C., Delahunt E., Holden S. (2017). Treatment and prevention of acute and recurrent ankle sprain: an overview of systematic reviews with meta-analysis. *British Journal of Sports Medicine*.

[6] Chen E. T., Borg-Stein J., Mcinnis K. C. (2019). Ankle sprains: evaluation, rehabilitation, and prevention. *Current Sports Medicine Reports*.

[7] Xiang A., Cheng K., Shen X., Xu P., Liu S. (2017). The immediate analgesic effect of acupuncture for pain: a systematic review and meta-analysis. *Evidence Based Complement Alternative Medicine*.

[8] Patil S., Sen S., Bral M. (2016). The role of acupuncture in pain management. *Current Sports Medicine Reports*.

[9] Kim T. H., Lee M. S., Kim K. H. (2014). Acupuncture for treating acute ankle sprains in adults. *Cochrane database of systematic reviews*.

[10] Suo L. W. Q. (2014). Study on the effect of acupuncture treatment of acute ankle sprain. *Contemporary Medicine Forum*.

[11] Du W. B., Bao G. A., Quan R. F. (2014). Impacts on analgesia and detumescence in ankle sprain treated with acupuncture at Xiaojie point combined with tendon-regulation manipulation. *Chin Acup Moxib*.

[12] Li K. (2016). *Clinical Study on the Curative of Contralateral Acupuncture on Acute Sprain of Ankle*.

[13] Zou S. Q., Li B. Q., Pan H. L. (2016). Clinical observation on acupuncture combined with sunshang emplastrum for acute ankle sprain. *Inner Mongolia Journal of Traditional Chinese Medicine*.

[14] Pei C. Q., Wei Y. (2017). Analysis of the efficacy of joint-corresponding point selection plus exercise acupuncture in treating acute ankle sprain. *Shanghai J Acu-Mox*.

[15] Wu C. Q., Chen M. (2017). Clinical observation on juci acupuncture combined with massage for acute ankle sprain. *Journal of Clinical Acupuncture and Moxibustion*.

[16] Wu S. C., You M. C., Yu P. (2018). Acupuncture combined with plaster immobilization for acute ankle sprain. *Asia-Pacific Traditional Medicine*.

[17] Li S., Zhang Y. Z., Liu J. Y. (2018). Analysis of therapeutic effect of acupuncture on patients with acute ankle sprain. *Clinical Journal of Traditional Chinese Medicine*.

[18] Moher D., Liberati A., Tetzlaff J., Altman D. G. (2009). Preferred reporting items for systematic reviews and meta-analyses: the PRISMA statement. *PLoS Medicine*.

[19] Koivu H., Kohonen I., Mattila K., Loyttyniemi E., Tiusanen H. (2017). Long-term results of scandinavian total ankle replacement. *Foot Ankle International*.

[20] De-Regil L. M., Peña-Rosas J. P., Fernández-Gaxiola A. C., Rayco-Solon P. (2015). Effects and safety of periconceptional oral folate supplementation for preventing birth defects. *Cochrane Database Systems Review*.

[21] Higgins J. P. T., Thomas J., Chandler J. (2019). *Cochrane Handbook for Systematic Reviews of Interventions Version 6.0*.

[22] Ma L. L., Wang Y. Y., Yang Z. H. (2020). Methodological quality (risk of bias) assessment tools for primary and secondary medical studies: what are they and which is better?. *Military Medical Research*.

[23] Yu J. (1999). A report of dimethyl sulfoxide gel, ice compress and acupuncture for treating 30 patients with ankle sprain. *Chinese Journal of Sports Medicine*.

[24] Yu J. (1999). Observation on the effect of acupuncture combined with dimethyl sulfoxide gel for ankle sprain. *Natural Science Journal of Hainan University*.

[25] Jiao H., Wang H. (2004). Clinical observation of sprain of the ankle joint treated with acupuncture and moxibustion under TDP irradiating. *Acta Academiae Medicinae CPAPF*.

[26] Wang X. L. (2005). Observation of the effect of electroacupuncture for lateral ankle ligament sprain. *Modern Journal of Integrated Traditional Chinese and Western Medicine*.

[27] Hao H. M., Wang X. (2006). Clinical observation of acupuncture combined with Chinese medicine for 63 patients with ankle sprain. *Journal of Shanxi College of Traditional Chinese Medicine*.

[28] Wu Z. S. (2007). *Clinical Study and Evaluation of the Efficacy of Blood-Pricking Therapy for Acute Ankle Sprain*.

[29] Ni X. P., Li Y. J. (2010). Observation of Clinical Efficacy of needing xiaojie point for sprain of ankle joints. *Inner Mongolia Journal of Traditional Chinese Medicine*.

[30] Sun C., Ju Y. Y. (2011). Evaluation on the effect of acupuncture in the acute ankle sprain induced by football. *Modern Preventive Medicine*.

[31] Zhang L., Zhang Y. P. (2011). Treating 90 cases of acute ankle sprain of students by combination therapy. *Clinical Journal of Chinese Medicine*.

[32] Kaminski T. W., Hertel J., Amendola N. (2013). National athletic trainers’ association position statement: conservative management and prevention of ankle sprains in athletes. *The Journal of Athletic Training*.

[33] van den Bekerom M. P., Struijs P. A., Blankevoort L. (2012). What is the evidence for rest, ice, compression, and elevation therapy in the treatment of ankle sprains in adults?. *The Journal of Athletic Training*.

[34] Park J., Shim J., Kim S. (2017). Application of massage for ankle joint flexibility and balance. *The Journal of Physical Therapy Science*.

[35] Miake-Lye I. M., Mak S., Lee J. (2019). Massage for pain: an evidence map. *Journal of Alternative and Complementary Medicine*.

[36] Carter D., Amblum-Almer J. (2015). Analgesia for people with acute ankle sprain. *Emergency Nursing*.

[37] Wang Y. H., Zeng K. W. (2019). Natural products as a crucial source of anti-inflammatory drugs: recent trends and advancements. *Traditional Medicine Research*.

[38] Ryu H. H., Kang J. C., Namgung U., Kim S. Y., Park J. Y. (2020). Anti-inflammatory effects of modified buyang huanwu decoction. *Evidance Based Complementary Alternative Medicine*.

[39] Zheng K., Chen Z., Sun W. (2018). Hei-gu-teng zhuifenghuoluo granule modulates IL-12 signal pathway to inhibit the inflammatory response in rheumatoid arthritis. *Journal of Immunology Research*.

[40] Chen T., Zhang W. W., Chu Y. X., Wang Y. Q. (2020). Acupuncture for pain management: molecular mechanisms of action. *American Journal of Chinese Medicine*.

[41] Choi D. C., Lee J. Y., Lim E. J. (2012). Inhibition of ROS-induced p38MAPK and ERK activation in microglia by acupuncture relieves neuropathic pain after spinal cord injury in rats. *Experimental Neurology*.

[42] Chen T. W., Yin Y., Zhang R., Ma W. Z. (2018). Fire-needle acupuncture intervention relieves ankle-joint inflammatory reactions possibly by down-regulating serum TNF-*α* and anti-cyclic citrullinated peptide antibody levels in collagen-induced Arthritis.Rats. *Zhen Ci Yan Jiu*.

