# Supplementary material for: Efficacy and Safety of Acupuncture Therapy for Patients with Acute Ankle Sprain: A Systematic Review and Meta-Analysis of Randomized Controlled Trials
Source: Evid Based Complement Alternat Med. 2020 Oct 16;2020:9109531. doi: 10.1155/2020/9109531 (PMC7585670; doi:10.1155/2020/9109531)
Supplement: Supplementary Materials — Detailed search strategy is shown in the Supplementary Material. [file 9109531.f1.docx]

Detailed search strategy:

PubMed:

#1: ankle[Title/Abstract] OR ankle[MeSH Terms]

#2: (sprains OR sprain OR strains OR strain OR injury OR injuries)[Title/Abstract]

#3: (acupuncture OR electroacupuncture)[Title/Abstract] OR acupuncture[MeSH Terms]

#4: (randomized OR randomly OR controlled OR trial)[Title/Abstract]

#5: #1 AND #2 AND #3 AND #4

Embase:

#1: ankle:ab,ti OR 'ankle'/exp

#2: sprains:ab,ti OR sprain:ab,ti OR strains:ab,ti OR strain:ab,ti OR injury:ab,ti OR injuries:ab,ti

#3: acupuncture:ab,ti OR electroacupuncture:ab,ti OR 'acupuncture'/exp

#4: randomized:ab,ti OR randomly:ab,ti OR controlled:ab,ti OR trial:ab,ti

#5: #1 AND #2 AND #3 AND #4

Cochrane library:

#1: (ankle):ti,ab,kw OR MeSH descriptor: [ankle] explode all trees

#2: (sprains):ti,ab,kw OR (sprain):ti,ab,kw OR (strains):ti,ab,kw OR (strain):ti,ab,kw OR (injury):ti,ab,kw OR (injuries):ti,ab,kw

#3: (acupuncture):ti,ab,kw OR (electroacupuncture):ti,ab,kw OR MeSH descriptor: [acupuncture] explode all trees

#4: (randomized):ti,ab,kw OR (randomly):ti,ab,kw OR (placebo):ti,ab,kw OR (tiral):ti,ab,kw

#5: #1 AND #2 AND #3 AND #4

Web of Science:

#1: TOPIC: (ankle) Indexes=SCI-EXPANDED Timespan=All years

#2: TOPIC: (sprains) OR TOPIC: (sprain) OR TOPIC: (strains) OR TOPIC: (strain) OR TOPIC: (injury) OR TOPIC: (injuries) Indexes=SCI-EXPANDED Timespan=All years

#4: TOPIC: (randomized) OR TOPIC: (randomly) OR TOPIC: (placebo) OR TOPIC: (trial) Indexes=SCI-EXPANDED Timespan=All years

#5: #1 AND #2 AND #3 AND #4

China National Knowledge Infrastructure:

(FT=脚踝 OR FT=脚部 FT=踝关节) AND (FT=扭伤 OR FT=拉伤 OR FT=损伤 OR FT=受伤) AND (FT=针刺 OR FT=针灸) AND (FT=试验 OR FT=观察 OR FT=随机 OR FT=疗效评价)

Wanfang Digital Periodicals:

(全部=脚踝 OR 全部=脚部 OR 全部=踝关节) AND (全部=扭伤 OR 全部=拉伤 OR 全部=损伤 OR 全部=受伤) AND (全部=针刺 OR 全部=针灸) AND (全部=试验 OR 全部=观察 OR 全部=随机 OR 全部=疗效评价)

Chinese Science and Technology Periodicals database:

U=(脚踝 OR 脚部 OR 踝关节) AND U=(扭伤 OR 拉伤 OR 损伤 OR 受伤) AND U=(针刺 OR 针灸) AND U=(试验 OR 观察 OR 随机 OR 疗效评价)
